# 

**Published:** 1831-04

**Authors:** 


### MONTHLY LIST OF MEDICAL BOOKS.

[*Medical Works cannot be entered on this List unless a copy be sent for the purpose, the titles of Books having frequently been sent to us as published which have not been published for weeks, or even months, after.*]

Professional Morality in 1831; or, the Lawyer's Defence of Medical Quackery: in which John St. John Long's Discoveries are examined, and his Claims to the Confidence of the British Public are criticised. Second Edition. By a Graduate of the University of Edinburgh, and a Master of Surgery and Arts.—8vo. pp. 80. London: Wilson, 1831.

A keenly written pamphlet. The practice and principles of St. John Long are ably discussed, and most bitterly, but justly, condemned. The "Member of the Inner Temple," who has recently undertaken the defence of Long, receives, from the eloquent pen of "A Graduate" a well-merited castigation; and we fear that some of the "regular" members of our own profession must writhe with the "pricking of their conscience" at many of the strictures contained in the last section of the pamphlet, under the head of the "Causes and Cure of Quackery."

Change of Air, or the Pursuit of Health; an Autumnal Excursion through France, Switzerland, and Italy, in the Year 1829: with Observations and Reflections on the Moral, Physical, and Medicinal Influence of Travelling-Exercise, Change of Scene, Foreign Skies, and Voluntary Expatriation. By James Johnson, M.D., Physician Extraordinary to the King.—8vo. pp. 294. London: Highley and Underwood, 1831.

Explanation of the Anatomical Atlas of Dr. M. J. Weber, Professor at the Royal Prussian University, Frederick William, at Bonn. Part II.—London: Schloss, 1831.

In this part of Weber's splendid work, the following organs are beautifully illustrated and correctly described: the Structure of the Lungs; Brain and Medulla Spinalis; Cerebrum; Cerebellum; Nerves of the Head and Neck; Nerves of the Chest and Abdomen; Nerves of the Uterus; the Arteries and Veins of the Diaphragm and Abdominal Organs. In order to render the minute structure of various parts of the human body clearly intelligible to the student, magnified views are given; and the natural size is also represented. By this judicious expedient, the minute structure of many parts, (of the lungs, for example,) will be perfectly comprehended without difficulty. We are indebted to Mr. Schloss for the publication of many other valuable works, and anatomical engravings, and these, of the celebrated Weber, form a very important and useful addition to the number.

An Address to the Medical Profession upon the Neglect of the Studies of Physiology and Morbid Anatomy. By R. Wade, Member of the Royal College of Surgeons, and Lecturer on Morbid Anatomy, &c.—8vo. pp. 16. Wilson, 1831.

An Appendix to the Popular Summary of Vaccination, comprising Experiments with Smallpox Virus upon the Cow, and further Remarks on the Practice of Cowpox. By John Marshall, Esq., Surgeon, District Vaccinator to the National Vaccine Establishment, &c.—8vo. pp. 38. London: Underwood, 1831.

This little essay contains some useful information upon the subjects indicated in the titlepage. As a proof of the general respect which is entertained for Mr. Marshall's opinions on vaccination, we may state, that his former work has been translated into French, and republished in America. It appears from the experiments detailed by Mr. Marshall in this "Appendix," that the "constitution of the cow possesses an innate power" of resisting the specific action of smallpox: inoculation with variolous matter, performed on the udder of cows, produced only "a temporary spot of inflammation."

**Cases of Lithotrity, or Examples of the Stone cured without Incision; followed by a Description of the first Symptoms of the Disease. By Baron Heurteloup, Doctor of the Faculty of Medicine of Paris.—8vo. pp. 54. London: Underwood, 1830.**

**Lecture introductory to a Course of Clinical Surgery, delivered to the Students of the Glasgow Royal Infirmary, by M. S. Buchanan, M.D., Member of the Faculty of Physicians and Surgeons, Glasgow, and one of the Surgeons to the Royal Infirmary, &c. (Not published.)**

### NOTICES.

*Communications have been received from* Mr. Edwin Long, Mr. Wa[illegible]e, &c.

